# Disseminated Intravascular Coagulation as the Initial Presentation of Metastatic Prostate Adenocarcinoma

**DOI:** 10.7759/cureus.14845

**Published:** 2021-05-04

**Authors:** Daniel J Greenberg

**Affiliations:** 1 Internal Medicine, Westchester Medical Center, Valhalla, USA

**Keywords:** disseminated intravascular coagulation, prostate cancer, thrombocytopenia

## Abstract

Disseminated intravascular coagulation (DIC) can be caused by cancer. However, it is rare to be the presenting sign of malignancy. The manifestations of bleeding or thrombosis in DIC depend on the balance of the fibrinolytic system. This case centers on a 52-year-old male who presented with DIC and spontaneous bruising, and no obvious cause for DIC. He was found to have metastatic prostate adenocarcinoma. DIC related to solid tumors typically have an indolent course and is only apparent on laboratory analysis. Further, DIC with excessive fibrinolysis in prostate cancer is associated with lower median survival. Treatment involves treating prostate cancer, and supportive treatment with blood products. Epsilon-aminocaproic acid may have a role in life-threatening bleeds, while prophylactic heparin treatment can be given for DIC associated with thrombosis.

## Introduction

The estimated incidence of disseminated intravascular coagulation (DIC) in prostate cancer is 13-30%. However, the clinical signs of DIC are only present in 0.4-1.65% of prostate cancer, and therefore, most of them are asymptomatic [1]. There are few cases of prostate cancer patients presenting initially with bleeding events, who are later found to have DIC secondary to prostate cancer [1-3]. In our case, we highlight a patient who presented with thrombocytopenia and spontaneous bruising, labs indicative of DIC, and found to have metastatic prostate adenocarcinoma.

## Case presentation

A 52 year-old male with history of heart failure with reduced ejection fraction and hypertension initially presented to an outside hospital with generalized fatigue and back pain. He was found to be thrombocytopenic, but records did not indicate the platelet count. He also experienced 20 pounds of unintentional weight loss in the last month, and no urinary complaints. His father had prostate cancer. On his discharge from the outside hospital, and eight days prior to admission to our hospital, his platelet count was 51 k/mm^3^, with a normal white blood cell count and hemoglobin. He was discharged on dexamethasone presumably for suspected autoimmune thrombocytopenia, and told to follow up with a hematologist. At an outpatient hematology visit, his platelet count was noted to be lower (unknown value), and he developed new ecchymosis on his right calf, so he was referred back to the emergency department at an outside hospital.

He was then transferred to our institution for further evaluation. He was afebrile, pulse 77 beats per minute, blood pressure 138/99 mm Hg, and saturating 96% on room air. Physical examination was unremarkable except for right posterior calf ecchymosis. Lab results (Table 1) raised concern for DIC. ADAMTS-13 (a disintegrin and metalloproteinase with a thrombospondin type 1 motif, member 13) level was normal.

**Table 1:** Laboratory Data Laboratory values on the day of admission, day 5 after admission (and before chemotherapy was given), and on the day of discharge. “X” indicates no lab result for that date. INR: international normalized ratio, PTT: partial thromboplastin time, AST: aspartate transaminase, ALT: alanine aminotransferase.

Test	Day 0 (day of admission)	Day 5 (before chemotherapy)	Day of discharge
White blood cell count (k/mm^3^)	23.7	15.7	18.0
Hemoglobin (g/dL)	14.1	11.4	7.8
Platelets (k/mm^3^)	30	9 (nadir)	32
Creatinine (mg/dL)	1.22	0.94	1.06
Lactate dehydrogenase (U/L)	2961	924	1859
Ferritin (µg/L)	8245.6	5372.5	X
D-Dimer (ng/mL)	35,013	>128,000	X
Fibrinogen (mg/dL)	62	381	284
Fibrin degradation products (µg/mL)	>40	X	X
INR	1.21	1.09	1.15
PTT (sec)	23.0	27.4	27.0
AST (U/L)	215	40	62
ALT (U/L)	43	33	26

**Figure 1:**
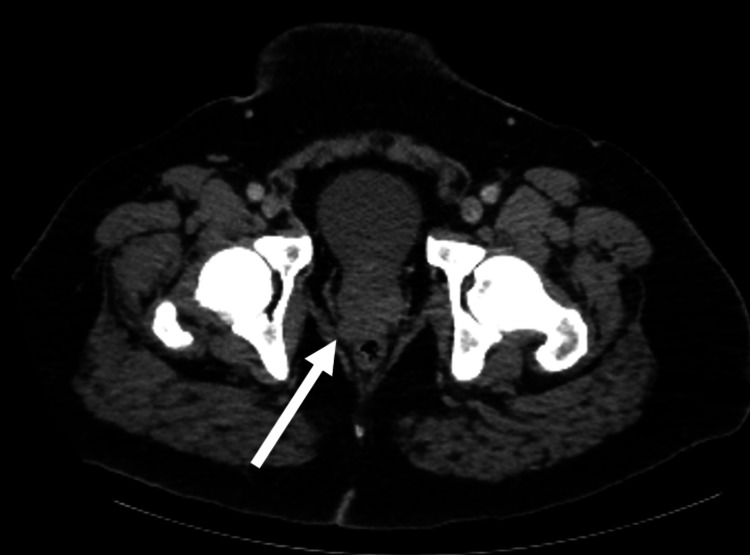
Axial CT scan of pelvic region There is mild bulging along the right posterior prostate base and seminal vesicle (white arrow) which may reflect the primary tumor.

**Figure 2:**
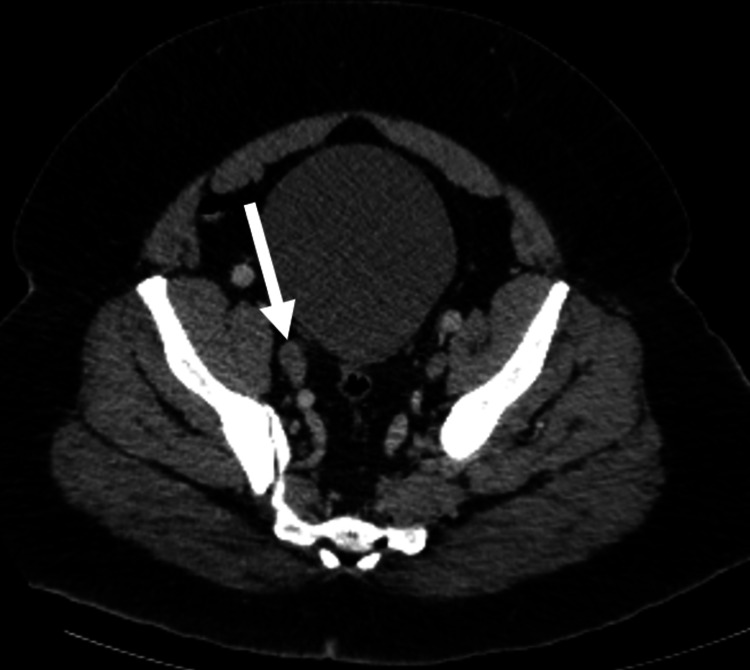
Axial CT scan of the pelvic region A mildly enlarged 12.6 mm right-sided pelvic lymph node is seen (white arrow).

Peripheral smear did not show any schistocytes. The infectious workup was negative. Computer tomography (CT) of the thorax/abdomen/pelvis with contrast was initially read as unremarkable. Due to continued uncertainty in the etiology of the DIC at this point, a bone marrow biopsy was performed and consistent with metastatic prostatic adenocarcinoma. Prostate-specific antigen was >500 ng/mL. His initial CT scan was reviewed again after the bone marrow biopsy, indicating mild bulging of the prostate base and seminal vesicle (Figure 1), as well as a mildly enlarged right-sided pelvic lymph node (Figure 2).

Bone scan showed increased uptake in right tenth rib and bilateral femoral diaphysis concerning metastasis. He received multiple supportive platelet transfusions over the course of his hospitalization to maintain the platelet level >10 k/mm^3^. He was started on leuprolide and docetaxel and discharged with plans to continue chemotherapy outpatient.

## Discussion

DIC is caused by diffuse intravascular activation and consumption of coagulation factors, which may lead to thrombosis and/or bleeding. The overall incidence of clinically overt DIC in solid tumors is 7%. Possible mechanisms of solid tumor-associated DIC include expression of tumor factor which can then activate coagulation factors, or by the expression of fibrinolytic proteins [1,4]. Further, DIC in cancer is usually less fulminant, more chronic, and subclinical with mostly normal coagulation parameters, until platelets are exhausted and bleeding occurs. When solid tumor patients with DIC do get complications, they are typically thrombotic more often than hemorrhagic in nature [4]. There are three subtypes of DIC based on the degree of fibrinolysis. These include DIC with suppressed fibrinolysis, balanced fibrinolysis, and enhanced fibrinolysis [5]. Our patient is considered to have DIC with enhanced fibrinolysis due to the excessive activation of the fibrinolytic system, as manifested by his significant thrombocytopenia, low fibrinogen, high fibrin degradation products, and hemorrhage due to the inability to compensate.

The presence of prostate cancer metastasis may be a significant risk factor in the development of DIC with excessive fibrinolysis. In one retrospective study of 42 patients with prostate cancer who developed DIC with excessive fibrinolysis, 40 of them had metastatic disease. Most occurred without an inciting factor; only three were provoked after surgical prostate manipulation. Further, the overall median survival for these patients 42 patients was only four weeks, versus 26 weeks if DIC resolved without needing supplemental blood products [6]. An earlier larger prospective study of DIC in solid tumors by Sallah et al. also showed reduced survival if DIC was present. If the patient had stages 1 and 2 diseases and DIC, they had a median survival of 16 months, compared to 44 months in those without DIC. Further, if they had advanced disease and DIC, median survival was 9 months compared to 14 months in patients without DIC [7].

Treatment for DIC involves reversing the cause. Sepsis, malignancy, obstetric disorders, and severe trauma may all trigger DIC. For prostate cancer-induced DIC, treatments include androgen deprivation therapy, chemotherapy, and radiation. In the interim, to manage complications, supportive treatment is recommended [8]. In patients with DIC and active bleeding, platelets should be maintained above 50,000, and fresh frozen plasma can be given. If there is active bleeding with low fibrinogen despite these supportive measures, cryoprecipitate can be given. In patients with prothrombotic or subclinical (i.e., balanced fibrinolysis) subsets of DIC, heparin anticoagulation can be considered prophylactically [9]. Epsilon-aminocaproic acid, a synthetic inhibitor of the plasmin-plasminogen system, may also have a role in life-threatening bleeding, according to a case report. It was successfully used to treat massive hemothorax in metastatic prostate cancer-induced DIC with hyperfibrinolysis [10].

## Conclusions

Our case highlights the importance of maintaining a high index of suspicion for malignancy in cases where the cause of DIC is unknown, and when non-invasive workup is initially negative. There should be a low threshold to pursue more invasive diagnostics. Doing so may help mitigate the increased morbidity and mortality associated with these patients. Patients should ideally be treated for their underlying malignancy to resolve the DIC. In the interim, supportive management is recommended.
